# A Shortened Barnes Maze Protocol Reveals Memory Deficits at 4-Months of Age in the Triple-Transgenic Mouse Model of Alzheimer's Disease

**DOI:** 10.1371/journal.pone.0080355

**Published:** 2013-11-13

**Authors:** Aida Attar, Tingyu Liu, Wai-Ting Coco Chan, Jane Hayes, Mona Nejad, KaiChyuan Lei, Gal Bitan

**Affiliations:** 1 Department of Neurology, University of California Los Angeles, Los Angeles, California, United States of America; 2 Brain Research Institute, University of California Los Angeles, Los Angeles, California, United States of America; 3 Molecular Biology Institute, University of California Los Angeles, Los Angeles, California, United States of America; University of Queensland, Australia

## Abstract

Alzheimer's disease is a progressive neurodegenerative disease that manifests as memory loss, cognitive dysfunction, and dementia. Animal models of Alzheimer's disease have been instrumental in understanding the underlying pathological mechanism and in evaluation of potential therapies. The triple transgenic (3×Tg) mouse model of AD is unique because it recapitulates both pathologic hallmarks of Alzheimer's disease - amyloid plaques and neurofibrillary tangles. The earliest cognitive deficits in this model have been shown at 6-m of age by most groups, necessitating aging of the mice to this age before initiating evaluation of the cognitive effects of therapies. To assess cognitive deficits in the 3×Tg mice, originally we employed a typical Barnes maze protocol of 15 training trials, but found no significant deficits in aged mice. Therefore, we shortened the protocol to include only 5 training trials to increase difficulty. We found cognitive deficits using this protocol using mainly measures from the probe day, rather than the training trials. This also decreased the effort involved with data analysis. We compared 3×Tg and wild-type mice at 4-m- and 15-m of age using both the original, long training, and the short training paradigms. We found that differences in learning between 3×Tg and wild-type mice disappeared after the 4^th^ training trial. Measures of learning and memory on the probe day showed significant differences between 3×Tg and wild-type mice following the short, 5-training trial protocol but not the long, 15-training trial protocol. Importantly, we detected cognitive dysfunction already at 4-m of age in 3×Tg mice using the short Barnes-maze protocol. The ability to test learning and memory in 4-m old 3×Tg mice using a shortened Barnes maze protocol offers considerable time and cost savings and provides support for the utilization of this model at pre-pathology stages for therapeutic studies.

## Introduction

Learning and memory deficits are relatively difficult to assess compared to other phenotypes, and although there is an abundance of papers describing cognitive deficit assessment in models of AD, replicating these studies *de novo* based on the literature often is challenging. Our comprehensive literature search resulted in Barnes maze [1] protocols with high variability of training periods, ranging from 4 d [2] to 15 d [3]. In addition, the age by which particular animal models of AD display cognitive deficits varies substantially, not only among models, but also in a particular model tested by different groups [2], [4], [5], [6], [7].

In addition to these challenges, assessing cognitive deficits in animal models is quite costly. For example, a new researcher embarking on assessment of learning and memory in a mouse model of AD using the Barnes maze at 8-m of age, who is paying animal *per diem* costs and minimum wage to a technician should expect to pay approximately $30,500 to establish the technique in their laboratory [Barnes maze – ∼$2500; video hardware and behavior detection software – ∼$8,000; aging animals – ∼$1.25/day for 8-m for 60 mice = $18,000; minimal colony maintenance, running an 8 d protocol and then analyzing 8 d of recorded behavior – ∼250 h paid at minimum wage ($8.00 in California) = $2000], in addition to the cost of obtaining and breeding the mice and many smaller but numerous expenses required for establishing a working system. If the mice need to be aged to an older age, as in the case of the 3×Tg model [8], which according to the literature often is used at 10-m of age or older to show convincing deficits [9], [10], [11] compared to control wild-type (WT) animals, the costs increase substantially.

The Barnes maze originally was developed by Carol Barnes for use with rats [1] to overcome the stress induced by swimming in the Morris water maze (MWM) [12], and later was adapted for mice [13]. During the task, animals are placed in the middle of a circular table containing holes around the edges and receive negative reinforcement, in the form of bright lights, an exposed environment, loud buzzing, and sometimes air jets [14], [15], motivating them to escape to a dark cage hidden underneath one of the holes. Similar to the MWM, the Barnes maze allows for evaluation of spatial reference memory and learning [16], but without inducing despair and anxiety that commonly are seen in the water maze in the form of floating and thigmotaxis [17], [18], [19]. At the same time, compared to the MWM, learning in the Barnes maze may be slow, and exploration high, due to the modest nature of the motivating stimuli [16]. Notwithstanding these differences between the two tests, many AD studies using mice have utilized the Barnes maze successfully to assess spatial memory [3], [20], [21], [22], [23].

Typical Barnes maze protocols consist of a habituation phase, in which the mouse is introduced to the environment and task, a training phase where the mouse is given numerous trials to learn the task, and a probe phase, typically performed following a 24-h delay, in which the mouse is tested for remembering what had been previously learned. Acquisition in the training phase typically is assessed as a decrease in latency and in the number of erroneous holes searched before finding the target hole, though not necessarily going into the escape cage. Entering the escape cage through the target hole often is not used as an end-point because, unlike in the water maze, the environment is not aversive enough to require immediate escape and mice may continue to explore after having identified the target hole. Other measures, such as path length or speed, also may be used [16], [24]. Long-term memory is evaluated in the probe phase, which occurs following training and a delay, by removing the escape cage and observing search behavior for a set amount of time. It is assumed that mice that remember the location of the escape cage will have a shorter latency to reach the previous location of the escape cage and will search fewer holes. Practically, this is measured as the time spent and holes searched (HS) in the target quadrant. A mouse with intact memory is expected to spend more than 25% (chance level) of their time in the target quadrant.

The 3×Tg mouse model of AD was developed in 2003 by the La Ferla group [8] and is unique in manifesting both amyloid plaques and neurofibrillary tangles in the brain. Thus, this model recapitulates the hallmark lesions of AD more closely than models that have only plaques or only tangles. The 3×Tg model, which harbors two familial AD mutations, APP(Swe) and PS1(M146V), and the tau(P301L) mutation found in frontotemporal dementia, has been integral in studies of the relationship between amyloid β-protein (Aβ) and tau [25], [26], and has been used to assess the role of intraneuronal Aβ [27], [28] and several potential therapies for AD [29], [30]. Studies by the LaFerla group on the cognitive deficits of this model have suggested that memory acquisition and retention were convincingly impaired starting at 4-m of age using either the MWM or the Barnes maze [4], [27]. However, other groups have not replicated deficits at this age. The youngest age at which groups other than LaFerla's have found deficits is 6-m of age using the MWM, WWWhich test, and/or nesting behavior [7], [31], [32]. Studies using the Barnes maze to assess the spatial reference learning and memory in the 3×Tg model found deficits at 4-m, 11-m, or 12-m of age [2], [5], [6]. These studies did not utilize the probe phase for measuring cognitive deficits. The one study in which deficits were found at 4-m of age was reported by the LaFerla group, who found that measures of latency showed progressive impairment with age but measures of error did not [2]. Frazer et al., who reported deficits at 11-m of age, did not detect deficits at 2-m or 6-m of age. In their study, all the animals were injected with a herpes simplex virus amplicon vaccine, thus a completely naïve control was only available for the 2-m group [5]. A study by Banaceur et al., in which deficits were found at 12-m of age, used only one age group, male mice, and only reported the measure of latency for training trials [6]. Potentially, the differences in Barnes maze protocols utilized in the above studies may have contributed to the different age of deficit onset observed.

Here we present an improved protocol, which allows testing learning and memory in the 3×Tg mouse model of AD using a short training paradigm at a young age, resulting in substantial saving of cost and time. To cut down the high costs, we constructed a homemade Barnes maze (<$300), devised a shortened training protocol consisting of only two training days, and used manual analysis of time and HS on only the probe day. Using this method, we found memory deficits in the 3×Tg model not only at 15-m of age but also at 4-m of age.

It is also our goal here to present some of the idiosyncrasies involved with this method. As we have been developing our protocol, we often encountered situations that either are not addressed in the literature or are not described in enough detail, and thus had to use our own judgment. We hope to lead by example by including our observations, such as the value of examining the range or median of data, which may not be directly results-related, but provide valuable insight and hope that these details are of value to other groups.

## Methods

### Animals

All procedures were compliant with the National Research Council Guide for the Care and Use of Laboratory Animals, and approved by the UCLA Institutional Animal Care Use Committee. 3×Tg and WT mice were bred at UCLA. Mice were housed 2–4 per cage under standard conditions, maintained on a 12-h dark and 12-h light cycle with *ad libitum* access to rodent chow and water, randomized, and handled under the same conditions by two investigators. Mixed-gender mice were tested at 4-m- and 15-m of age with n = 14–32 mice per group and a minimum of n = 7 of each gender per group.

### Barnes Maze

Barnes maze was administered to assess cognitive deficits in learning and memory of 3×Tg mice compared to the WT group. The maze was made from a circular, 13-mm thick, white PVC slab with a diameter of 48”. Twenty holes with a diameter of 1.75” were made on the perimeter at a distance of 1” from the edge. This circular platform was then mounted on top of a rotating stool, 35” above the ground and balanced.

The escape cage was made by using a mouse cage and assembling a platform and ramp 1.25” below the surface of the maze. The platform, made of a square petri dish, and ramp, made of laminated cardboard, were made out of plastic to be easily cleanable with 70% ethanol. The outside of the walls of the cage was covered with black paper to make the inside of the cage dark and thus attractive to the mice. The maze was placed in the center of a dedicated room and two 120 W lights were placed on the edges of the room facing towards the ceiling about 3/4 of the way up from the floor and about 3–5 feet away from the maze. Eight simple colored-paper shapes (squares, triangles, circles) were mounted around the room as visual cues, in addition to the asymmetry of the room itself. After testing each mouse, the cleaning of the quadrant of the maze around the target hole was alternated with cleaning the whole maze, using 70% ethanol. The maze was rotated clockwise after every 3 mice to avoid intra-maze odor or visual cues. All sessions were recorded using COP Security Monochrome CCD Camera (Model 15-CC20) and MyTV/x software (Eskape Labs).

The animals interacted with the Barnes maze in three phases: habituation (1 day), training (2–4 days in the short or long training paradigms, respectively; Table 1), and probe (1 day). Before starting each experiment, mice were acclimated to the testing room for 1 h. Then all mice (n = 2–4) from one cage were placed in individual holding cages where they remained until the end of their testing sessions. Holding cages were used during the experiment to control for potential artifacts that could result from housing some mice only two per cage, and remained alone while the other mouse was being tested, compared to other mice that were housed four per cage and therefore never were left on their own. Additionally, using holding cages prevented potential influence by mice that had already completed the test on the mice waiting for their turn. After all mice from one home cage completed testing for the day, they were placed back in their home cage together, the holding cages were cleaned, and the next set of mice was separated into individual holding cages.

**Table 1:** Comparison of short and long training paradigms.

	Training duration (min)	Probe trial duration (min)	# of training trials	# of training days	Total protocol time (days)*
Short paradigm	2	2	5	2	4
Long paradigm	2	2	15	4	6

* Total time does not include the day of rest between training and probe phases.

On the habituation day, the mice were placed in the center of the maze underneath a clear 3,500-ml glass beaker for 30 s while white noise was played through a sound system. Then, the mice were guided slowly by moving the glass beaker, over 10–15 s to the target hole that leads to the escape cage. The mice were then given 3 min to independently enter through the target hole into the escape cage. If they did not enter on their own during that time, they were nudged with the beaker to enter. Getting the mice to enter the escape cages is key in “showing” them that the escape cage exists and gives them practice in stepping down to the platform in the cage. The mice were allowed to stay in the escape cage for 1 min before being returned to the holding cage. Once all animals had completed the 1-session habituation, they were all returned to their home cage.

In the training phase, mice were placed inside an opaque cardboard cylinder, 10” tall and 7” in diameter, in the center of the Barnes maze for 15 s. This allowed the mice to be facing a random direction when the cylinder was lifted and the trial began. At the end of the holding period, a buzzer was turned on, the cylinder was removed, and the mice were allowed to explore the maze for 2 min (Table 1). If a mouse found the target hole and entered the escape cage during that time, the end-point of the trial, it was allowed to stay in the escape cage for 1 min before being returned to the holding cage. If it did not find the target hole, the mouse was guided to the escape hole using the glass beaker and allowed to enter the escape cage independently. If it did not enter the escape cage within 3 min, it was nudged with the beaker until it did. If a mouse still did not enter the escape cage after 1 min of nudging, it was picked up and manually put on the platform in the escape cage. Then it was allowed 1 min inside the escape cage before being returned to the holding cage. In all cases, the buzzer was turned off once the mouse entered the escape cage. This process typically took 5–7 min per mouse and was done with four mice at a time, providing a 20–30 min inter-trial interval. The total number of trials used was 5 for short training, 3 trials on training day 1 and 2 trials on training day 2, or 15 for long training with 3 trials on day 1 and 4 trials for days 2–4 (Table 1). During the training phase, measures of primary latency and primary HS were recorded. Primary latency was defined as the time to identify the target hole the first time, as mice did not always enter the hole upon first identifying it. HS was defined as nose pokes and head deflections over any hole. Primary HS was defined as the HS before identifying the target hole for the first time. Parameters were assessed by blinded observers. About 70% of the measures were randomly reassessed by a second blinded observer to identify potential inaccuracies. Differences between the two observers were insignificant in all cases. In all the cases in which two observers scored the raw data, their scores were averaged.

**Figure 1:**
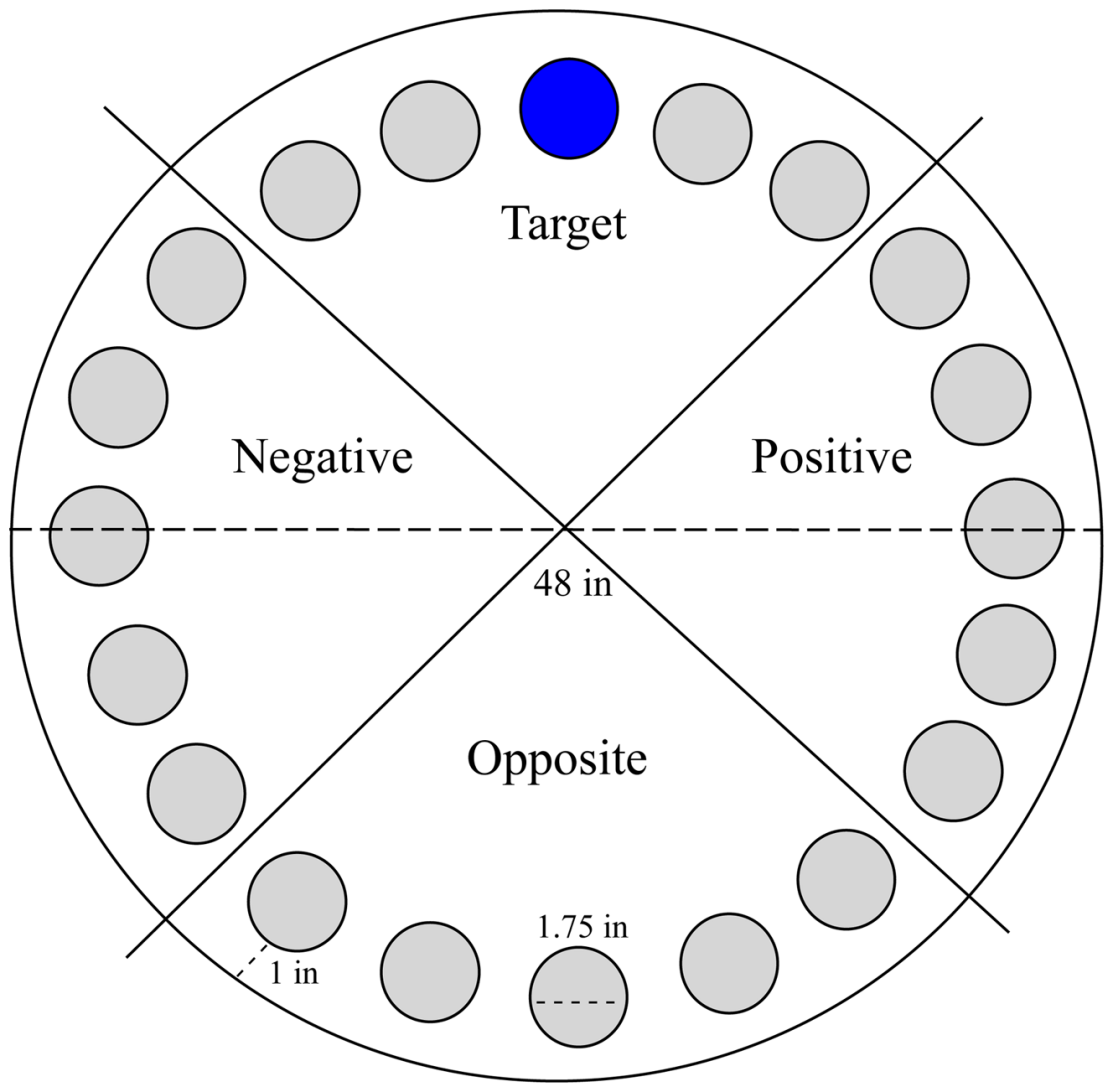
Barnes maze diagram with quadrants. The Barnes maze is made up of a circular platform, 48” in diameter, with 20 equally spaced holes around the periphery. The holes are 1” away from the edge and have a 1.75” diameter. The maze is divided into 4 quadrants labeled Target, Positive, Opposite, and Negative with the escape hole being in the center of the Target quadrant.

On the probe day, 48 h after the last training day, the escape cage was removed, mice were placed inside the opaque cylinder in the center of the maze for 15 s, the buzzer was turned on and the cylinder removed. Each mouse was given 2 min to explore the maze, at the end of which, the buzzer was turned off and the mouse was returned to its holding cage. During the probe phase, measures of time spent per quadrant and HS per quadrant were recorded. For these analyses, the maze was divided into quadrants consisting of 5 holes with the target hole in the center of the target quadrant (Fig. 1). The other quadrants going clockwise from the target quadrant were labeled: positive, opposite, and negative.

An observation of potential value is that 8 mice at 15-m of age and 2 mice at 4-m of age fell off the Barnes table during the training trials on the first day of training. Typically, they fell through one of the holes by attempting to extend their view and not off the edge. Initially, they were placed back in the center of the maze and the study continued. However, these mice were excluded from data analysis. It remains to be determined what this observation may signify.

### Statistics

Data are shown as means ± standard error of the mean (SEM). Statistical analysis was performed using Prism 6.0c (GraphPad, La Jolla, CA). Student's unpaired *t*-test and 2-way repeated measures ANOVA followed by Fisher's Least Significant Difference *post-hoc* analysis were used for probe day and training trials data, respectively. The level of significance was set at *p*<0.05.

## Results

### Training trials – Comparison of 15 trials versus 5 trials

We began our use of the Barnes maze because we were interested in assessing the cognitive benefits of small molecule aggregation inhibitors for Alzheimer's disease therapy [33]. Based on our extensive literature search on the Barnes maze in AD models, we developed a 15-training trial protocol and following its execution, found that either our 3×Tg mice did not have cognitive deficits compared to WT mice or the test was not sensitive enough to detect the deficits.

**Figure 2:**
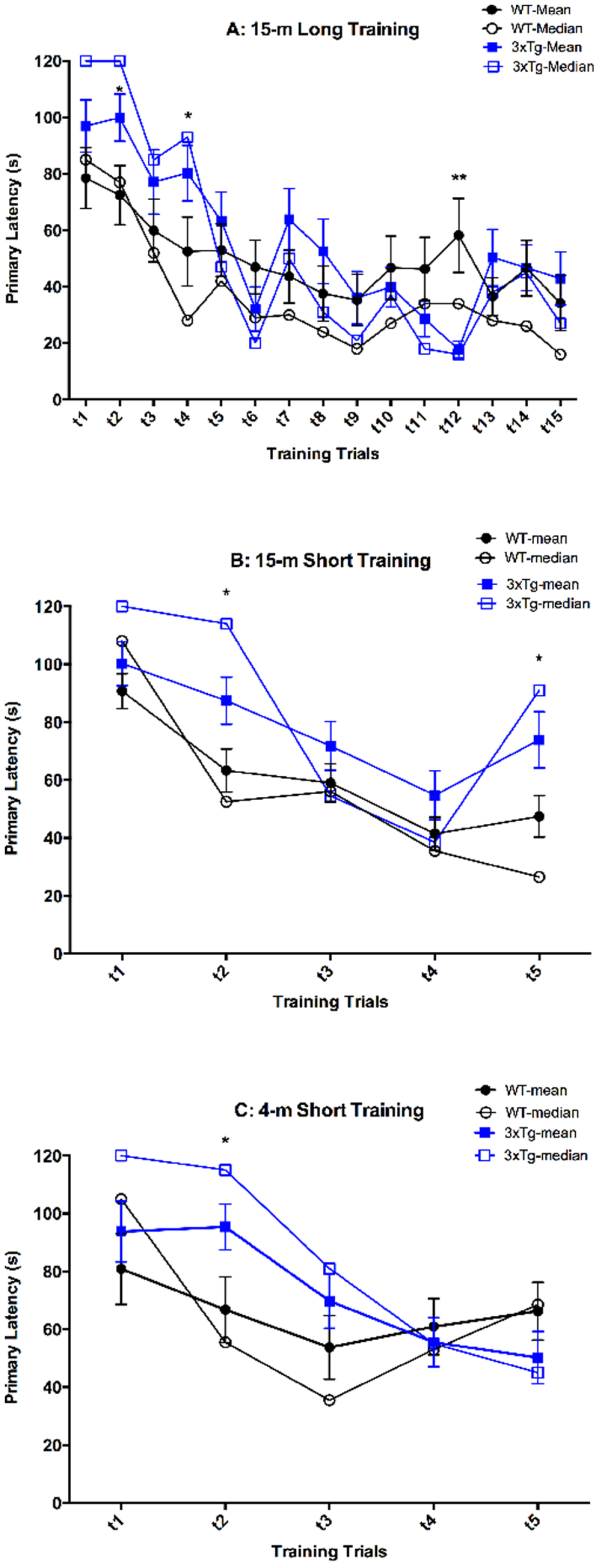
Primary latency of training trials shows group differences only in first 4 trials. A) Primary latency, out of 120 s, for 15-m old wild-type (WT) or triple transgenic (3×Tg) mice receiving 15 training trials (WT n = 32, 3×Tg n = 24). Mean and median values given for comparison. Primary latency over 5 training trials for 15-m old (B; WT n = 15, 3×Tg n = 15) and 4-m old (C; WT n = 14, 3×Tg n = 17) mice. *p<0.05, **p<0.01 compare mean values of WT and 3×Tg.

Our analysis of the training day latencies in the initial long-training paradigm, which included 15 training trials showed that consistent differences in latency between the WT and 3×Tg groups existed only in the first 4 trials followed by stochastic values in the remaining trials, especially for the 3×Tg group (Fig. 2A). Repeated-measures ANOVA with *post-hoc* analysis showed significant differences on trials 2, 4, and 12, yet examination of the entire trend suggested that the difference observed on trial 12 likely was coincidental. Thus, we hypothesized that much of the training after trial 4 was redundant and leading to elimination of cognitive difference between the groups. Thus, we developed a shortened Barnes maze paradigm to test this hypothesis.

Short training, consisting of 5 trials, of 15-m old mice showed significant differences between WT and 3×Tg mice on trials 2 and 5 (Fig. 2B). Latency measures in 4-m old mice administered short training showed a significant difference between groups on trial 2 (Fig. 2C). Based on these data, we argue that latency data from training days is not robust enough to establish meaningful differences and is greatly influenced by the high variability of the system, resulting in potentially false positive data. Many studies examine, or even only examine, differences in latency or HS between groups on training days. Though these measures can illuminate differences between groups, the differences often occur on only one or two of many training trials. Our study suggests that relative to the value gained, the time and effort required for analysis of training days is not an efficient use of resources.

Because in the long-training paradigm the latency means for trials 5–15 were highly variable within each group, we asked whether the range of latencies might offer additional information. The range of latencies for WT and 3×Tg mice in trials 5–15 was 34–58 s and 18–64 s, respectively. This suggested that the 3×Tg mice actually reached a shorter average latency (on trial 12) than the WT mice, which seemed counterintuitive. However, when the raw latency values for the mice were evaluated, it became evident that this observation was due to an artifact created by using the arithmetic mean population descriptor (i.e., the average of the population). This causes larger numbers to have a larger weight even though a more reasonable analysis would give each animal's latency value the same weight. Thus, we posit that the median is a better population descriptor in this situation. Comparison of the 3×Tg mean and median curves shows very similar results. Notably, the median latency values for trial 1 and 2 are increased relative to the mean values. Comparison of the WT mean and median curves shows a general drop in latencies on trials 4 and later. Thus, the range of median trial latencies for WT and 3×Tg mice in trials 5–15 changes to 16–42 and 16–50 s, respectively, supporting the conclusion that the WT mice learned as well as the 3×Tg mice did.

### Probe day – Comparison of long versus short training in 15-m old mice

**Figure 3:**
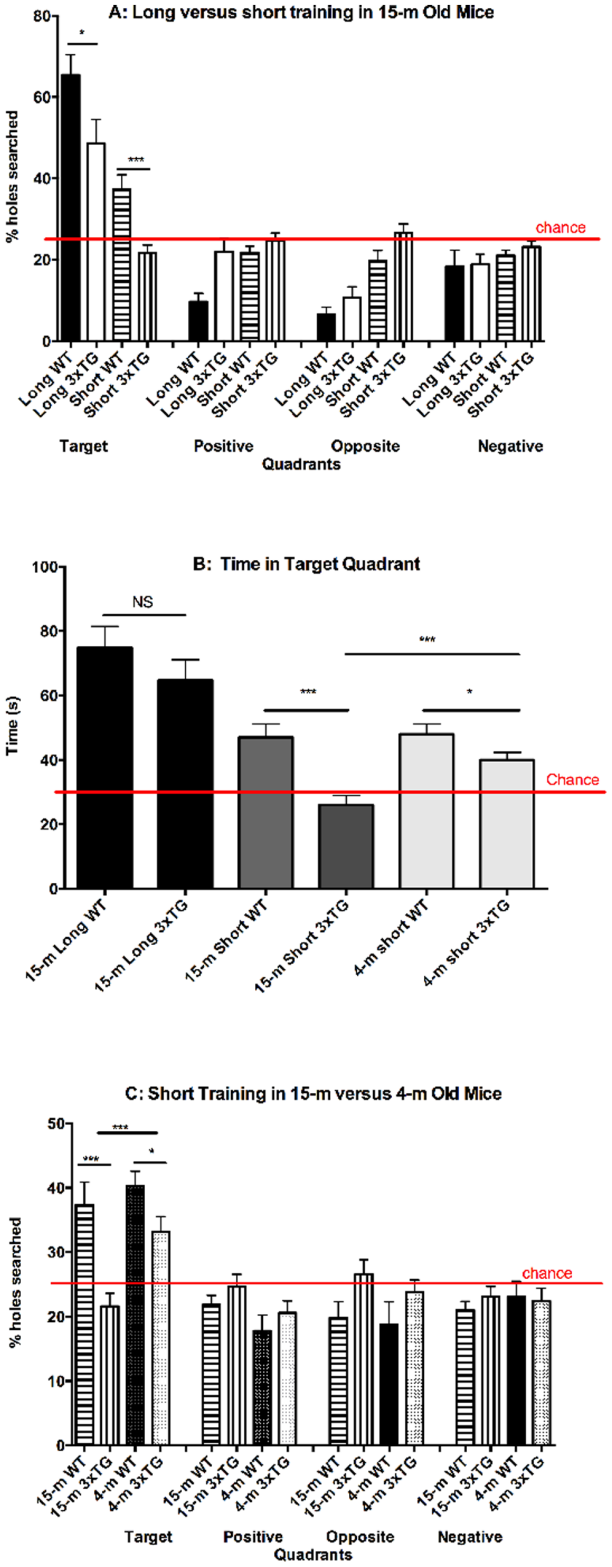
Percent holes searched and time in target quadrant show short training can resolve cognitive deficits. A) Percent holes searched, on probe day, in each of four quadrants by 15-m old wild-type or triple transgenic mice receiving either short or long training. Chance level of holes searched in each quadrant is 25%. B) Time (s) spent in the Target quadrant by all 6 groups of mice. Chance amount of time spent per quadrant is 30 s out of 120 s. C) Percent holes searched in each of four quadrants by WT or TG and 15-m or 4-m old mice receiving short training. *p<0.05, ***p≦0.001 compare WT and 3×Tg.

Initially, we used the long training paradigm to compare 15-m old WT and 3×Tg mice. Using this paradigm, the differences between the 3×Tg mice and the WT mice in the number of HS and time spent in the target quadrant, which measure the ability of the mice to remember the general location of the escape hole on probe day, were small (Fig. 3). Though the 3×Tg mice showed significantly lower percent HS in the target quadrant compared to the WT mice (WT 65.4±4.9%, 3×Tg 48.6±5.9%; *p*<0.05, Fig. 3A), the time spent in the target quadrant was not significantly different between groups (described below, Fig. 3B). Moreover, the % HS in the target quadrant for both groups was prominently above a chance level of 25% (Fig. 3B) indicating that learning and long-term memory were intact, albeit less efficient in the 3×Tg group.

To test if the results reflected over-training of the mice, we shortened the number of training sessions from 15 to 5. Using this short-training paradigm, a more pronounced difference was observed in % HS in the target quadrant between the WT and 3×Tg mice (WT: 37.3±3.5%, 3×Tg: 21.6±2.0%; *p*<0.001; Fig. 3A). Importantly, 3×Tg mice receiving short training did not search in any quadrant at levels higher than chance suggesting that they did not remember which quadrant contained the escape cage. The difference between the WT and 3×Tg in % HS on probe day indicated a deficit in memory retrieval rather than in learning for both the long and short training paradigms because all groups demonstrated learning, by a decrease in latency, of the target hole on training days (Fig. 2).

Similar results were observed using measures of time (Fig. 3B) or % time (data not shown) spent in each quadrant on probe day. Long training of 15-m old mice resulted in similar values, which were significantly above chance (30 s) for both WT and 3×Tg mice. In contrast, short training resulted in highly significant differences between the WT and 3×Tg groups. The 3×Tg spent near chance levels of the time in each quadrant.

Previous studies have shown that the 3×Tg mouse model presents not only with gender differences in brain pathology [33], [34], but also in behavior [4], [32]. Thus, we evaluated the effect of gender on the behavior of the different groups. We did not find significant differences in % HS in the target quadrant on probe day between males and females of either genotype with either training paradigm (data not shown).

### Probe day – Comparison of young (4-m) versus old (15-m) mice in the short-training paradigm

Age is a highly important factor in studies related to AD. Not only is age a major determinant of phenotype and disease progression, but also in studies of animal models, the age of the animals has a substantial effect on the study cost. Following the development of an improved, short paradigm allowing observation of robust, significant differences between old (15-m of age) WT and 3×Tg mice, we asked whether such differences also could be observed in young mice. To answer the question, we trained and tested 4-m old WT and 3×Tg mice using the short-training paradigm.

Following short-training, both 4-m old WT and 3×Tg mice performed above chance levels in the % HS in the target quadrant on the probe day (Fig. 3C). The % HS in the target quadrant by the 4-m old WT mice (40.2±2.3%) was similar to that of the 15-month old WT mice (37.3±3.5%), suggesting that the age difference was not a significant determinant of memory retention in the WT group. In contrast, the 4-m old 3×Tg mice displayed 54% better ability to remember the target quadrant than their 15-m old counterparts (4-m 33.2±2.4%, 15-m 21.6±2.0%; *p*<0.001), suggesting that in the presence of the transgenes, age was an important contributor to memory decline. Despite the improved memory of the young 3×Tg relative to the old 3×Tg mice, the difference between the 4-m old WT and 3×Tg groups still was statistically significant (WT 40.2±2.3%, 3×Tg 33.2±2.4%; *p*<0.05; Fig. 3C). Comparison of the time spent in the target quadrant between the WT mice at 15-m (47.0±4.2 s) and 4-m (48.0±3.2 s) showed similar values, whereas the 3×Tg mice show a larger difference of 35% with increasing age (15-m 26.0±2.9 s vs 4-m 40.0±2.4 s; *p* = 0.001). No effects of gender were found in the 4-m old WT or 3×Tg mice.

### The ‘Motivation’ factor

The motivating stimuli for any behavioral task often are of great importance. Many studies use food or water deprivation, or survival instinct (in the case of the MWM), to instigate the mice to perform the task. Other tasks use natural tendencies such as object- or environment-exploration and thus do not add stress on the animals, with the cost of a decrease in the task-instituted motivation. One potential weakness of the Barnes maze test may be the relatively mild aversive stimuli used to motivate the mice to find the escape cage.

Our data suggest that the total number of HS on probe day, regardless of quadrant, may be an indication of motivation. Fifteen-month old mice receiving long training, regardless of genotype, and 4-m old WT mice receiving short training, searched on average in 16–17 holes with a similar range — 1–37 holes and 8–35 holes for 15-m WT and 3×Tg, respectively, and 5–36 holes for 4-m WT mice. Interestingly, 15-m old mice receiving short training, regardless of genotype, and 4-m old 3×Tg mice receiving short training searched on average in 22–23 holes. The range of hole searched was substantially higher for these groups — 2–58 holes and 0–51 holes for 15-m old WT and 3×Tg, respectively and 8–48 holes for 4-m 3×Tg mice. One interpretation of these results is that the long training, and thus more experience with the task where no major threats are felt, in the 15-m old mice and the WT genotype in the 4-m old mice confers a feeling that the mouse is safe and decreases the anxiety and motivation to search for escape on the probe day.

**Figure 4:**
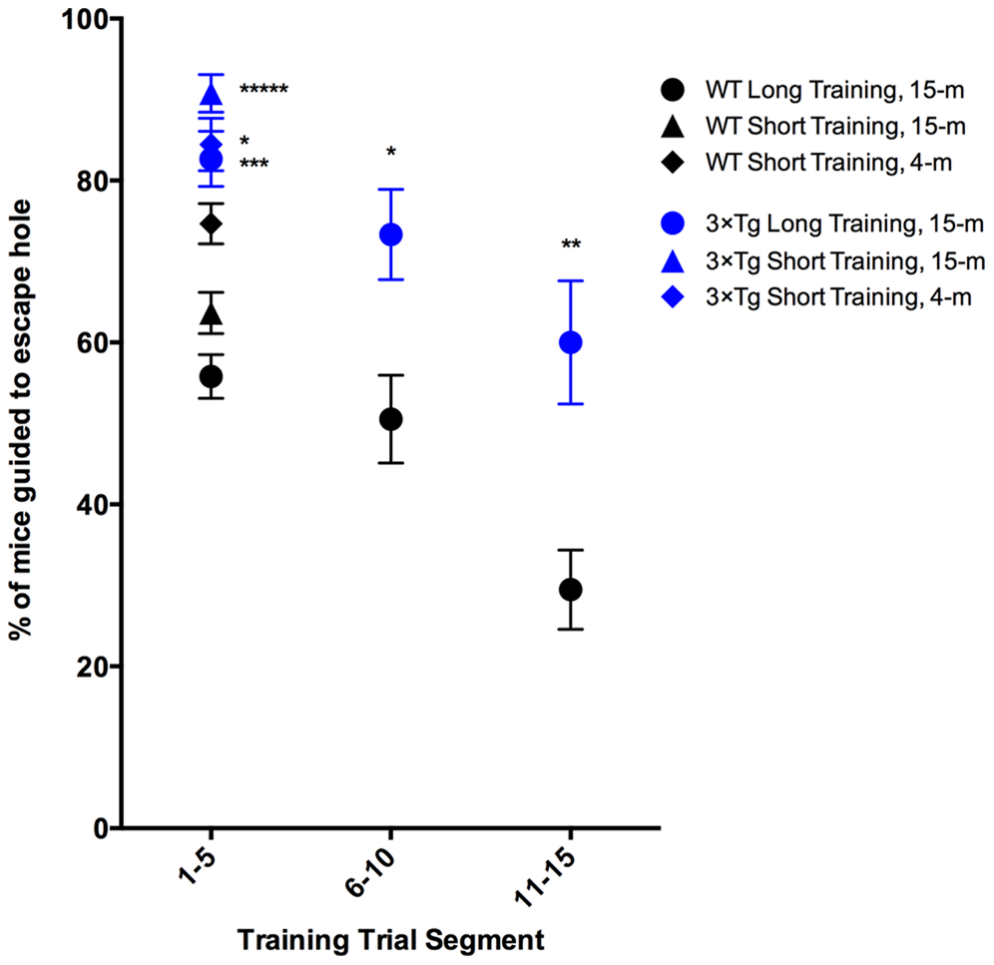
Percent of mice being guided to escape hole on training days decreased with training. Percent of mice being manually guided to the escape hole that did not independently enter in the allotted 2: trials 1–5, 6–10, and 11–15. *p<0.05, **p<0.01, ***p<0.001, ****p<0.0001 compare WT and 3×Tg.

Motivation also can be measured by the number of mice who needed to be guided to the escape hole during training days because they did not enter the escape hole on their own in the allowed time (Fig. 4). Notably, this does not suggest that the mice did not identify the escape hole on training days, only that they did not go into the escape hole. Typically, the measure of primary HS, rather than total holes searched before entering the hole, can be used to overcome the effect of low motivation to enter the escape cage on evaluation of learning. Fifteen-months-old mice receiving long training needed to be guided to the escape hole at the end of their allotted time on average for the first five trials 56% and 83% of the time for WT and 3×Tg, respectively. This value decreases to 51% and 73% for trials 6–10 and to 29% and 60% for trials 11–15 for the WT and 3×Tg mice, respectively. Fifteen-months-old mice receiving short training needed guidance to the escape hole on average for the total five trials 64% and 91% for WT and 3×Tg, respectively, and 4-m old mice receiving short training needed guidance 75% and 84% of the time for WT and 3×Tg, respectively. Three conclusions can be gleaned from these data. First, 3×Tg mice enter the escape cage on their own less often than WT mice, potentially indicating hypoactivity akin to AD-like apathy, as reported by Filali et. al. [35]. However, our analysis of total HS does not show a difference between 15-m old WT and 3×Tg mice when compared between similar training lengths. The difference seen between the 4-m old WT and 3×Tg also is not statistically significant. Second, the percentage of mice that entered the escape cage voluntarily increased with added training trials, indicating increased motivation to enter the escape cage. Lastly, age did not affect motivation to enter the escape cage in the 3×Tg mouse model. Thus, motivation in the Barnes maze task potentially can be separated from changes in cognitive function. Though these results regarding manual guidance of mice do not directly affect measures of learning, as the end-point is hole identification and not entering hole, analysis of percent of mice guided to the escape hole suggests that motivation to enter the escape cage is low in early trials, especially for 3×Tg mice.

## Discussion

### Data from probe day is superior to data from training days

Our study illuminates the higher value of probe-day measures over training-day measures by demonstrating a correlation between performance and age, amount of training, and presence of transgenes. Inspection of HS or time spent in the target quadrant (Fig. 3) shows the effects of extra training sessions on memory retrieval, the changes in cognition as a result of aging or the presence of transgenes, and several combinations thereof. These results are robust and are detectable following analysis of one 2-min trial per animal rather than the substantially longer and labor-intensive training-day analyses of 5 or 15 trials per animal. The decrease in data processing allows for a more accurate manual analysis, compared to tracking-software analysis, which is prone to recognition biases, such as different or sufficient body parts present in the target zone for a sufficient amount of time [36].

Comparisons of Fig. 2 panels A–C show that 3×Tg mice started with 120 s median latency in the first two trials, whereas WT mice started with 85–110 s median latency in the first trial and often improved by the second trail. All groups of mice showed shorter latencies over the next few trials and converged at either trial 4 or 5. The reduction in escape latency during the training trials is similar to learning curves in the hidden-platform MWM test. However, similar decreases in escape latencies have been observed in visible-platform MWM trials and cued Barnes maze studies [13], [36]. When the mice can see the platform, a decrease in latency likely is not due to spatial learning, but rather due to habituation to the environment eventuating in decreased anxiety and increased motivation to escape over repeated trials [36]. This theory is supported by our observation of a decrease in the number of HS on probe day with long training and the decrease in the need for manual guidance of mice to the escape hole during the last 10 training trials in the long-training paradigm (Fig. 4). A putative high level of anxiety at the start of the study seems to be especially prominent in the 3×Tg mice, which, compared to the WT mice, always started at a higher latency on trial 1. In addition, the 3×Tg mice showed significantly larger latencies on training-trial 2, compared to WT mice, regardless of age or training length (Fig. 2).

Increased anxiety in this model has been identified previously [37], [38]. Possibly, the consistent significant difference on trial 2 between the 3×Tg and WT mice suggests that the 3×Tg mice not only have higher anxiety levels, but also take longer to habituate to their environment. Our protocol consisted of a 30 s habituation phase and guidance to the escape hole to “show” the mice that escape existed. This habituation could be extended to potentially mitigate some of the initial anxiety due to the novel environment. Possibly, if both 3×Tg and WT mice had an extended habituation time, both groups might start at a lower initial latency and might still be different from each other or the extended habituation could serve to close the starting gap between the 3×Tg and WT. We theorize that a combination of both scenarios may be the most likely because of the elimination of observable cognitive deficits found with overtraining, as discussed in the next section. As the longer training closed the gap in performance between the WT and 3×Tg mice and did not just increase both groups' time in the target quadrant (Fig. 3B), it is probable that additional habituation would both decrease initial latency values for both groups and close the gap between them.

Significant differences in latency between WT and 3×Tg also were observed following the divergence, on trial 5 in the 15-m short training group (Fig. 2B) and on trial 12 in the 15-m long training group (Fig. 2A). However, considering the global trends of the data, specifically, the variability of the individual trial mean values and values for trials 10, 11, 13, and 14 in Fig. 2A, the high significance found on trial 12 appears to be a mere coincidence.

### Observing cognitive deficits depends on the difficulty of the task

Comparison of the long- and short-training paradigms (Fig. 3B) reveals that the number of training sessions affects directly the time spent in the target quadrant on the probe day, which indicates the ability of the mice to remember the location of the target hole. Importantly, our study shows that overtraining makes the probe day task too easy and results in elimination of observable cognitive deficits between WT and 3×Tg mice. Strong evidence for the high impact of overtraining is the fact that 4-m-old WT mice who received short training had less % HS in the target quadrant (40%, Fig. 3C) than old 3×Tg who received long training (49%, Fig. 3A), indicating that with sufficient training, the memory impairment caused by age and presence of the three dementia-causing transgenes can be overcome. Similar results are seen with time spent in the target quadrant measure (Fig. 3B). An additional difference between our Barnes maze protocol and typical published protocols that may increase the difficulty of the task is that we allowed for a 48 h delay between the training trials and the probe day. This assumes that the amount of delay is related to the difficulty of the task by requiring more neural processing for consolidated learning and long-term memory.

Ideally, the number of training trials and delay time would be calibrated to result in a difficulty level that leads animals with expected memory deficits to spend only chance levels of time or of % HS in the target quadrant as was achieved in the 15-m 3×Tg group receiving short training (Fig. 3A, C). Chance-level behavior can be a useful additional indication of the difference between groups and can help reduce the probability that a particular measure, e.g., % HS in the target quadrant would show a difference whereas another measure, e.g., time in target quadrant, would not show a difference between the 3×Tg and WT groups, as was the case for the 15-m old mice receiving long training. The possibility to carefully adjust the number of training trials and delay time and thus the difficulty of the task is an advantage of the Barnes maze and other learning tasks relative to tasks that solely rely on exploratory behavior.

To our knowledge, this is the first study other than those by LaFerla's group, to show cognitive deficits in the 3×Tg mouse model at 4-m of age. Presumably, this was achieved thanks to our optimization of the Barnes-maze training paradigm. Frazer et al. who showed deficits in the 3×Tg mice at 11-m of age, but not at 2-m or 6-m of age, using the Barnes maze used 3 training trials for 1 day at 2-m, 6-m, and 11-m [5]. They reported measures of distance, errors, and latency averaged over the 3 trials during the training day yet did not perform a probe trial. A trend in the data of Frazer et al. suggested that latency was higher at 6-m than at 2-m. However, high variability, possibly due to a relatively small number of animals (n = 6) per group, might have prevented reaching statistical significance in that study. It is possible that Frazer et al. did not detect deficits at a younger age because they only tested learning, not long-term memory, and the 3×Tg mice show learning over the training trials in most studies. In addition, the values over the 3 trials were averaged together, which can mask an initial deficit. LaFerla and colleagues [2], who showed deficits in the 3×Tg mice at 4-m of age used 4 training trials per day for 4 days. They performed a long-term memory trial at 24-h and 7-d after the fourth day of training. However, the escape cage was present during these retention trials. Their study reported higher latency values for 3×Tg mice compared to WT mice on training days starting at 2-m of age. In addition, they found significantly longer escape latency at the 24-h memory retention test but not at the 7-d retention test in the 2-m old 3×Tg mice. The average number of HS in the target quadrant was not significantly different between 3×Tg and WT mice at 2-m of age. Thus, not all measures showed deficits at 2-m of age. The differences between 3×Tg and WT mice, by measures of training trial escape latency, 24-h and 7-d retention trial escape latencies, and HS in the target quadrant became significantly different at 4-m of age. Our data suggest that Clinton et al. might have been able to detect consistent significant differences between the 3×Tg and WT groups at 2-m if less training had been used.

### More versatile models are necessary to break the cycle of failed drugs

The ability to identify cognitive deficits and to evaluate therapeutic means for rescuing or preventing these deficits is a fundamental tool needed for therapy development in the AD field. Having this ability in young mice has several advantages. First, little time needs to be spent aging mice leading to cost reduction and allowing for a shorter experiment-planning time resulting in more possible leads being tested. Second, being able to test the cognition of animal models of AD before amyloid plaque deposition in the brain allows testing prevention, which likely will be more advantageous than treatment approaches as actual therapy for AD. For example, Das et al. treated Tg2576 mice with a γ-secretase inhibitor from 4-7-m of age, prior to the onset of the exponential increase in Aβ deposition. A much larger decrease in Aβ levels (60%) was observed in this group compared to treatment from 7-10-m (34%) or 12-15-m (no effect) of age. Importantly, in all cases, the mice were sacrificed and their brains analyzed when they reached age 15-m [39]. Clinical trials of Aβ lowering drugs, such as γ-secretase inhibitors or immunotherapeutics, in symptomatic patients have been unsuccessful, prompting concerns that such strategies may be of limited efficacy when used in symptomatic patients with AD compared to prevention at pre-symptomatic stages [40]. On the other hand, prophylactic administration of drugs without a good screening procedure for AD, which begins 10–20 years before the onset of symptoms [41], [42], may be cost-prohibitive. A reasonable compromise is a hypothetical treatment paradigm, in which people may be treated preventatively, for example, for several months every 5 years beginning at age 40. If human data were to echo the findings by Das et al. [39], such a strategy could result in a delay of pathology progression and potentially the onset of disease by years. We recognize that transgenic mouse models are not perfect proxies for human disease, as they lack neurodegeneration and timing of appearance of biological and functional pathology cannot always be directly translated. Nonetheless, these models are essential for identification of leads and detection of early memory deficits offers the benefits of testing multiple leads with time and cost savings, as discussed above.

Several studies have examined shortened training paradigms in the MWM [36], [43]. In contrast, to our knowledge, this is the first systematic analysis of the effect of the number of training trials, and comparison of training versus probe days, on the sensitivity of the Barnes maze to detect cognitive deficits, and its validation in young transgenic AD mice. Our study provides compelling evidence for using a short-training paradigm and for inclusion of a probe trial that can produce robust distinctions between 3×Tg and WT mice. These factors and the validation of cognitive deficits in 4-m old 3×Tg may bring us one step closer to finding disease-modifying therapy for AD.
